# Match Duration and Number of Rallies in Men’s and Women’s 2000–2010 FIVB World Tour Beach Volleyball

**DOI:** 10.2478/v10078-012-0068-7

**Published:** 2012-10-23

**Authors:** José Manuel Palao, David Valades, Enrique Ortega

**Affiliations:** 1 Faculty of Sport Science- University of Murcia, Spain.; 2 Faculty of Sport Science- University of Alcala, Spain.

**Keywords:** sport, training, evolution, rules, beach volleyball

## Abstract

After the 2000 Olympic Games, the Fédération Internationale de Volleyball (FIVB) modified the scoring system used in beach volleyball from side-out to a rally point system. The goal was to facilitate the comprehension of the game and to stabilize match duration. The purpose of this study was to assess the duration and number of rallies in men’s and women’s beach volleyball matches (2000–2010 FIVB World Tour). Data from 14,432 men’s matches and 14,175 women’s matches of the 2000–2010 World Tour were collected. The variables studied were: match duration, total rallies per set and match, number of sets, team that won the set and match, type of match (equality in score), and gender. The average match duration in beach volleyball is stable, ranging from 30 to 64 minutes, regardless of the number of sets, the stage of the tournament (qualifying round or main draw), or gender. The average number of rallies per match were 78–80 for two-set matches and 94–96 for three-set matches. Matches from the main draw are more balanced than matches from the qualifying round. More balanced matches (smaller point difference between teams) have longer durations. It is not clear why there is no relationship between the number of rallies and match duration. Future studies are needed to clarify this aspect. The results can serve as a reference to guide beach volleyball training (with regard to duration and number of rallies) and to help understand the effect of the rule change.

## Introduction

Beach volleyball has much developed in recent years at all levels (participation, sponsors, number and level of championships, TV coverage, audience, prize money, etc.). With the goal of contributing to beach volleyball development, after the 2000 Olympic Games, the Fédération Internationale de Volleyball (FIVB) modified the scoring system from side-out to the rally point system. The aim of this change was to simplify the comprehension of the rules for the general public and make the matches’ duration more predictable and controllable (Marques et al., 2010; Pérez-Turpin et al., 2009). This last aspect was in order to adapt it to the demands of TV, as a spectacle sport. A similar change was made by the FIVB two years earlier in indoor volleyball. One of the effects of the change in the scoring system was a reduction in the average match duration from 96.1 minutes to 66.86 minutes (Ureña et al., 2000). After the change, a volleyball match’s duration is shorter and more stable, as it has less variation. In beach volleyball, the FIVB changed the scoring system, the competition format (from one set to best of three sets), and the court size (9 x 9 m to 8 x 8 m) at the same time. The reduction in the court size was done to increase continuity in the game. According to the studies found, the effect of these changes has resulted in a decrease in match duration from 41.6 ± 10.2 minutes to 35.5 ± 9.9 minutes (Giatsis, 2003). At the same time, the rule changes have also changed the way various technical actions are executed such as the serve, attack, and block, as well as their efficacies (Giatsis and Tetzis, 2006; Ronglan and Grydeland, 2006).

In relation to match duration, the study by Giatsis (2003) showed the short-term effect of the change in scoring system on match duration and the number of rallies. That study compared the 582 matches of the women’s 2000 World Tour with the 582 matches of the women’s 2001 World Tour (main draw). In the conducted review of literature, no more studies were found with regard to the evolution of match duration and the number of rallies through time (long-term effect) with the rally point system. The information about match characteristics is important as a first step to design training sessions (to assess the characteristics of the sport) and to understand the effect of the rule changes. Therefore, this paper assesses the long-term effect of the change in scoring system and court size on match duration and number of rallies and its evolution during the last decade. The purpose of this study was to assess the duration and number of rallies in men’s and women’s beach volleyball matches (2000–2010 FIVB World Tour).

## Material and Methods

Data from 14,432 men’s matches (36.0 % from qualifying matches and 64.0 % from the main draw) and 14,175 women’s matches (31.3 % from qualifying matches and 68.7 % from the main draw) of the 2000–2010 World Tour organized by the Fédération Internationale de Volleyball (FIVB) were collected. Data were obtained from the FIVB’s website (www.fivb.com/en/beachvolleyball). The variables studied were as follows: match duration, total rallies per set and match, number of sets, team that won the set and the match, type of match, established through the point difference between teams (very balanced, 0–4 point difference; balanced, 5–8 point difference; unbalanced, 9–12 point difference; and very unbalanced, more than 12 point difference), and gender. The type of match was established from the data analysis. The point difference between matches was calculated, and from these data the percentiles were used to establish the differences between categories. Unfinished matches (due to sanction, injured player, etc.) were not considered for this study (0.75 % of the matches). Descriptive and inferential analyses of the data were done. To analyze the different variables in the study, a one-factor analysis of variance between the various specific positions was used, as well as the Sheffe Post Hoc analysis. All of the statistical analyses were done with a level of significance set at p ≤ 0.05. All statistical analyses were conducted using the SPSS statistical package (version 18.00, Institute Inc., Cary, NC, USA).

## Results

With regard to match duration, for men, the average was 42 ± 14 minutes for both two-set and three-set matches, and for women, the average was 39 ± 18 minutes for two-set matches and 40 ± 17 minutes for three-set matches. For two-set matches, the minimum duration was 5 minutes in a men’s qualifying round match, and the maximum duration was 100 minutes in a women’s main draw match. For three-set matches, the minimum duration was 17 minutes in both a women’s qualifying round match and a main draw match, and the maximum duration was 152 minutes in a women’s main draw match. Significant differences were found between duration of qualifying and main draw matches for men (p < 0.001). Qualifying matches were an average of five minutes longer. No differences were found in the duration of women’s competitions between qualifying and main draw matches. In Table 1 and Table **2**, the evolution of match duration through the time period that was analyzed is shown. When comparing the first five years and the second five years, there was a significant increase in match duration. They were 2.8 minutes longer for men’s two-set matches (p < 0.001) and 3.2 minutes longer for men’s three-set matches. No significant differences were found for women’s matches. The same tendency was observed in the qualifying round and main draw matches.

With regard to rallies played per match, for men, the average was 80 ± 13 rallies per twoset match and 96 ± 16 rallies per three-set match, and for women, the average was 78 ± 14 rallies per two-set match and 94 ± 36 rallies per three-set match. For two-set matches, the minimum number of rallies played was 46 rallies for women’s matches in the qualifying round and the maximum was 142 rallies in a women’s qualifying round match.

For three-set matches, the minimum number of rallies found was 53 rallies in a women’s match in the main draw, and the maximum number was 168 rallies in a men’s match in the main draw. Significant differences were found in the number of rallies played in the qualifying matches and the main draw matches for both men and women (p < 0.001).

Matches in the main draw were an average of 16.9 rallies longer for men and 22.3 rallies longer for women. In Table 3 and Table 4, the evolution of match duration through the period of time that was analyzed is shown. When comparing the first five years and the second five years, a significant reduction by 10 rallies for men (p < 0.001) and 19.8 rallies for women (p < 0.001) in two-set matches and an increase by 7.8 rallies for men (p<.001) and 16.5 rallies for women (p < 0.001) in three-set matches was observed. The same tendency was observed in both qualifying matches and main draw matches.

**Figure 1:**
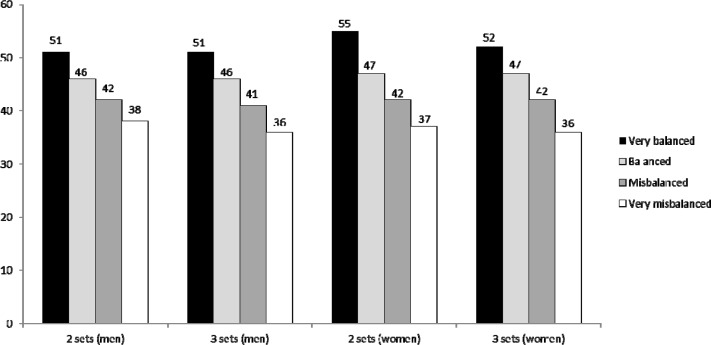
Average duration of matches (minutes) in relation to the type of match (balance in the score)

The more balanced matches were significantly longer (p < 0.001). This tendency was observed in qualifying and in main draw matches in both the men’s and women’s competitions. These average differences are presented in Figure 1 for two-set and three-set matches for men and women. Between 31 and 36 % of the matches needed the third set to determine the winning team, and in the third set no significant differences were observed between the final outcome of the match and having won the first set. In 49.3 – 50.2 % of the cases, the team that won the first set also won the third set. In the qualifying rounds of the women’s competition, this value was lower at 47.7 %, although this difference was not significant.

## Discussion

The purpose of the paper was to study the long-term effect of the rule changes (scoring system and court size) on match duration and number of rallies. From a general perspective, the results show that the change in the scoring system has resulted in an average match duration and number of rallies that are stable regardless of the number of sets, the stage in the tournament (qualifying rounds or main draw), or gender. Specifically, in relation to match duration, it was surprising to find practically the same values for match duration for two-set and three-set matches (39 - 42 minutes). The data show that 90 % of the matches, regardless of the number of sets played, are between 30 and 64 minutes. These values differ from data found by Giatsis (2003). The differences are likely due to the size of the sample and the way data were selected (one tournament). The data from this study were from all the matches played in the World Tour during the 2001–2010 seasons. For the men’s competition, an increase in match duration has been observed in the last five years (an increase of approximately 2–3 minutes). Future studies should monitor this tendency to assess whether men’s matches keep lengthening or stabilize. From the data of this study, it is not possible to establish the reason for this increase. It could be due to an increase in teams’ balance, which leads to more time-outs, time lost in the matches (extra rest time), or more continuity in the game.

The number of rallies per match were 78–80 for two-set matches and 94–96 for three-set matches. Matches from the main draw had more rallies than matches from the qualifying round. These results probably indicate more equality between teams at this stage of competition. Two aspects that were found about the number of rallies are difficult to explain because they should affect match duration but they did not. The first of these aspects is that in the last five seasons a reduction in the number of rallies played has been observed for two-set matches though there has also been an increase in the number of rallies played in three-set matches. This should mean that two-set matches were shorter and three-set matches were longer. However, match duration was almost the same regardless of the number of sets. The second aspect is that when the data were divided by the equality of the teams in the match (determined by the difference in points at the end of a match), matches that were more balanced were significantly longer. This aspect did not affect the overall match duration either. A possible explanation for these results could be the type of match phase or complex involved in the matches or the strategies of the team during the matches (Pérez-Turpin et al., 2009). For example, with the rally point system, differences of six or seven points are difficult to recover because of the high success ratio of the side-out (Palao, 2004), so it is possible that losing teams risk a lot with their serve and their actions to try to recover the score difference and at the same time rest for the next set or match. The possible effect of the change in court size, which was done at the same time as the scoring system change, should also be considered. This aspect has changed the way beach volleyball is played (Giatsis and Tetzis, 2006; Ronglan and Grydeland, 2006), as well as the use, efficacy, and importance of the different technical actions, game phases, etc. (e.g. serve, block, defense phase, etc.). Specific studies to assess why these aspects do not affect the overall match duration are needed.

In relation to the number of sets and the final outcome, 66.4 – 68.6 % of the matches end in two sets. In beach volleyball, when a team wins the first set, in 83 – 84 % of the cases, that team wins the match. However, when the opponent wins the second set, the chance of winning the match does not depend on whether the team won the first or the second set.

The average match duration in beach volleyball was 39–42 minutes but varied from 30 to 64 minutes, regardless of the number of sets, stage of the tournament (i.e. qualifying round or main draw), or gender. Over the last five seasons in men’s competition, there has been a slight increase in match duration of 2–3 minutes. The number of rallies per match were 78–80 for two-set matches and 94–96 rallies for three-set matches. Matches from the main draw are more balanced than matches from qualifying rounds. A tendency for matches of two sets to have fewer rallies and matches of three sets to have more rallies is observed in the last five seasons. More balanced matches (i.e. smaller point difference between teams) have a longer duration. It is not clear why there is no relationship between the number of rallies and match duration.

## Conclusions

The results can serve as a reference to guide beach volleyball training (with regard to duration and number of rallies). The standard match duration for beach volleyball is between 30 and 64 minutes and involves between 78 and 96 rallies. Future studies are needed to study more in depth the reason for some of the results that were found (e.g. relationship between match duration, tendencies over the last five years, etc.). The differences between this study and previous studies show the need to study not only the short-term effect of rule changes but also the long-term effect. There is a need to accumulate enough data to see the effect and evolution of the sport after the rule changes. More studies are needed to contextualize beach volleyball because the changes done by the FIVB did not only involve a change in the scoring system but also a change in court size at the same time. Therefore, it is still not clear how this change has affected the sport of beach volleyball.

## Figures and Tables

**Table 1:** Match duration in men’s competition (World Tour seasons 2001 to 2010)

**Year**	**Two-set matches**					**Three-set matches**				

	**n**	**X**	**SD**	**P-5**	**P-95**	**n**	**X**	**SD**	**P-5**	**P-95**
2001	914	44	11	29	66	423	45	11	29	66
2002	475	39	9	27	56	273	40	10	27	57
2003	701	42	10	29	59,5	372	42	10	29	60
2004	824	42	10	29	62	409	42	10	28	60
2005	959	42	10	30	59	460	42	10	29	60
2006	894	43	10	29	62	445	44	11	29	64
2007	1048	45	10	31	63	563	45	10	31	63
2008	1169	40	18	31	63	589	39	18	31	65
2009	854	40	19	33	67	428	42	17	32	68
2010	932	40	20	33	67	472	41	19	34	65

Total	8770	42	14	30	63	4434	42	14	30	63

P-5: Percentile 5; P-95: Percentile 95

**Table 2:** Match duration in women’s competition (World Tour seasons 2001 to 2010)

**Year**	**Two-set matches**					**Three-set matches**				

	**n**	**X**	**SD**	**P-5**	**P-95**	**n**	**X**	**SD**	**P-5**	**P-95**
2001	767	38	18	31	62	313	38	17	29	60
2002	362	37	17	29	61	130	37	18	29	64
2003	896	39	19	30	64	353	40	19	30	66
2004	699	44	17	33	69	344	44	16	34	65
2005	913	40	17	31	64	436	41	16	32	65
2006	926	41	17	32	66	430	42	16	32	64
2007	1054	39	18	31	64,5	493	39	19	32	66
2008	1084	37	19	31	62	515	39	18	32	62
2009	890	39	17	31	63	437	39	16	32	62
2010	956	38	17	31	60	470	41	18	31	64

Total	8547	39	18	31	64	3921	40	17	31	64

P-5: Percentile 5; P-95: Percentile 95

**Table 3:** Rallies played in men’s competition (World Tour seasons 2001 to 2010).

**Year**	**Two-set matches**					**Three-set matches**				

	**n**	**X**	**SD**	**P-5**	**P-95**	**n**	**X**	**SD**	**P-5**	**P-95**
2001	914	83,2	15,9	66	113	423	87,9	16,5	68	114
2002	475	86,8	17,6	67	114	273	84,5	16,6	67	113
2003	701	86,3	17,2	67	112	372	83,1	17,9	67	113
2004	824	86,4	16,3	68	115	409	85,1	17,0	67	114
2005	959	84,4	17,2	66	113	460	84,3	18,5	67	114
2006	894	75,0	6,7	66	87	445	105,3	8,3	95	120
2007	1048	75,0	6,5	66	87	563	105,1	7,5	95	119
2008	1169	75,4	6,6	66	87	589	105,2	8,1	95	120
2009	854	75,2	6,6	65	87	428	104,8	7,4	95	120
2010	932	75,4	6,6	66	88	472	104,3	7,2	95	118

Total	8770	79,6	13,3	66	107	4434	96,3	16,2	70	118

P-5: Percentile 5; P-95: Percentile 95

**Table 4:** Rallies played in women’s competition (World Tour seasons 2001 to 2010)

**Year**	**Two-set matches**					**Three-set matches**				

	**n**	**X**	**SD**	**P-5**	**P-95**	**n**	**X**	**SD**	**P-5**	**P-95**
2001	767	81.3	16.3	63	108	313	83.7	16.3	63	112
2002	362	81.5	15.6	64	111	130	79.8	14.0	63	106
2003	896	81.7	15.9	64	111	353	82.2	17.0	63	111
2004	699	84.7	16.2	66	113	344	84.2	16.9	66	113
2005	913	83.2	16.3	64	110	436	84.8	17.0	64	113
2006	926	76.7	22.7	0	109	430	90.2	98.5	60	113
2007	1054	74.4	7.0	64	86	493	104.6	7.4	95	118
2008	1084	74.2	6.5	64	85	515	104.3	6.6	95	117
2009	890	74.0	6.6	64	84	437	104.7	7.1	95	117
2010	956	116.4	1299.2	65	85	470	103.4	7.4	93	117

Total	8547	78.0	14.2	64	106	3921	94.4	36.0	67	116

P-5: Percentile 5; P-95: Percentile 95
